# Time-series analysis for rapid event-related skin conductance responses

**DOI:** 10.1016/j.jneumeth.2009.08.005

**Published:** 2009-11-15

**Authors:** Dominik R. Bach, Guillaume Flandin, Karl J. Friston, Raymond J. Dolan

**Affiliations:** Wellcome Trust Centre for Neuroimaging, University College London, 12 Queen Square, London WC1N 3BG, United Kingdom

**Keywords:** Skin conductance, SCR, Galvanic skin response, GSR, Electrodermal activity, EDA, Convolution, Deconvolution, General linear model, Linear time invariant filter

## Abstract

Event-related skin conductance responses (SCRs) are traditionally analysed by comparing the amplitude of individual peaks against a pre-stimulus baseline. Many experimental manipulations in cognitive neuroscience dictate paradigms with short inter trial intervals, precluding accurate baseline estimation for SCR measurements. Here, we present a novel and general approach to SCR analysis, derived from methods used in neuroimaging that estimate responses using a linear convolution model. In effect, the method obviates peak-scoring and makes use of the full SCR. We demonstrate, across three experiments, that the method has face validity in analysing reactions to a loud white noise and emotional pictures, can be generalised to paradigms where the shape of the response function is unknown and can account for parametric trial-by-trial effects. We suggest our approach provides greater flexibility in analysing SCRs than existing methods.

## 1 Introduction

Skin conductance responses (SCRs) are peripheral indicators of sympathetic activation widely used in psychological and neuroscientific research (Boucsein, 1992). In some applications, SCRs constitute the primary outcome variable, for example when analysing the autonomic orienting reflex or conditioned anticipatory fear responses. In other fields, SCRs are used to quantify levels of arousal associated with emotional and cognitive processes. SCRs have an extended temporal profile. In many experimental situations involving rapid stimulus presentation, the evoked SCRs overlap. In particular, in the domain of cognitive neuroscience and functional neuroimaging, where rapid event-related designs are now commonplace, classical analytic methods relying on measuring each evoked response relative to a pre-event baseline are of limited utility.

To overcome this limitation, alternative methods have been proposed. Barry et al. (1993) attempted to correct the baseline by subtracting each SCR from an extension of the preceding SCR, using graphical tools. Lim et al. (1997) converted this idea into a numerically tractable problem by assuming a skin conductance response function with four parameters optimised for each individual response. The amplitude for this response is then scored individually as in classical analysis methods (Lim et al., 1999a,b; Williams et al., 2004). This method is limited by the number of overlapping responses such that when responses are close together, their parameters need to be estimated at the same time. It is also not clear under which circumstances such combined estimations have unique solutions. In contrast, Alexander et al. (2005) derived measures for individual SCRs by assuming an underlying sudomotor nerve signal that drives the SCR. The relationship between the two is described as a differential equation, integration of which yields the driver function. Peaks of the driver function are isolated, and individual SCRs are calculated from peaks in the estimated driver signal. Unlike more elaborate deconvolution methods (e.g. Wiener deconvolution), this approach does not explicitly model measurement noise, and must therefore cope with noise in the estimated driver signal. This is reduced by smoothing the driver function and applying ad hoc restrictions on the peak detection algorithm.

All of the aforementioned approaches share two main disadvantages with classical peak-scoring methods. Firstly, they do not make use of all available information, as only peak amplitude and latency, or the area under the curve, are analysed. Secondly, and more importantly, all three make implicit assumptions about the shape of the SCR that are not fully described in analytical terms and are therefore not testable. The method of Lim et al. (1997) comes closest to specifying an analytical response model. However, since different functions and parameters are used for each response, and later collapsed by analysing the amplitude value, this specification is lost.

Here, we describe a completely novel approach that compares the SCR time series as a whole to a predicted time series using a general linear convolution model. The method is akin to the analysis of event-related functional magnetic resonance imaging (fMRI) data, and shares with the latter the problem of a temporally extended response function. In the first part of this paper, we describe our overall approach and outline its assumptions. The model is then applied to unselected (i.e. representative) samples of SCR time series. In experiment 1, we test an invariance assumption on which the models rests and derive a canonical skin conductance response function. Experiment 2 demonstrates deconvolution for different ISIs and demonstrates the use of the canonical response function. In experiment 3, we further validate this model in the context of a more complex experiment involving SCRs to emotional pictures. To make the method widely accessible, we have created an open-source software suite for Matlab R2007 and upwards, named *SCRalyze*, freely available under the *GNU General Public License* and obtainable from *scralyze.sourceforge.net*.

## 2 Model description

The challenge addressed by our model is to separate, or deconvolve, overlapping SCRs evoked by different experimental manipulations. To render this problem uniquely solvable, we make simplifying assumptions (see appendix for a more formal description; see Friston et al., 1994 for an application of this idea in fMRI). We do not claim that SCR time series conform to these assumptions in any strict sense, but we demonstrate that the method is robust to violations of underlying assumptions. However, we explicitly describe these assumptions in order to render them testable and go on to demonstrate that they are sufficiently met with regard to practical implementation.

**Fig. 1:**
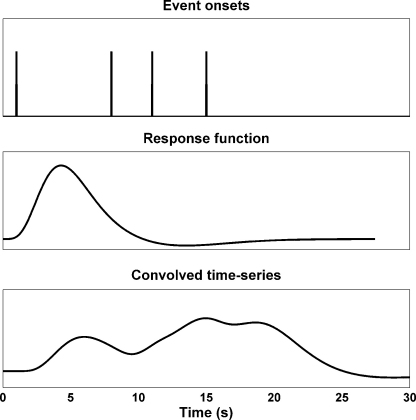
Convolved time-series: an example from functional magnetic resonance imaging (fMRI). Top: event onsets are specified as a stick, or delta function. Middle: the canonical hemodynamic response function is the most parsimonious response function used in fMRI research. Bottom: convolution of the event onset function with the response function results in a time series that reflects the overlay of single responses.

The assumptions are: (a) the shape of the response is constant within an individual and level of an experimental factor, although the amplitude can vary as a function of the input; (b) two overlapping responses constitute the sum of two single responses; (c) at some time-point after each response, the signal returns to zero, as approximated by high-pass filtering the signal and thereby removing slow changes in the skin conductance level (SCL). These assumptions can be formalised by positing that the filtered SCR time series is the output of a finite Linear Time Invariant (LTI) filter, given a specified input function (see Fig. 1). It turns out that a violation of these model assumptions leads to greater error terms and conservative testing.

### 2.1 Specifying the impulse response function

**Fig. 2:**
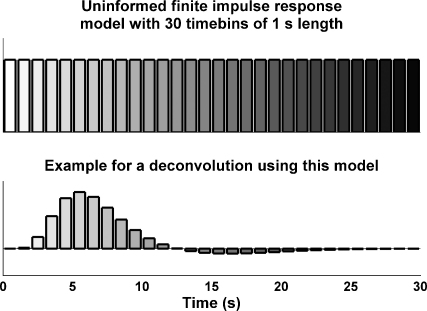
Basis set for an uninformed finite impulse response model, with 30 time bins of 1 s length. Each time bin codes a regressor across events. An idealised example for typical parameter estimates in fMRI is shown below. This procedure of estimating a response function is profoundly different from “averaging” responses, since during averaging, overlapping segments are accounted for several times.

The shape of the response is described by an impulse response function, assumed to be constant within each individual and level of experimental factor. This function needs to be specified and we consider two approaches for this specification. (a) The first is to assume that the response function is unknown and derive its shape from the data. This approach only makes assumptions about the duration of the response and models the finite impulse response filter with a number of boxcar functions covering the desired response length (see Fig. 2). This approach is often termed *FIR model* in fMRI research; in this paper we will call it an *uninformed finite impulse response filter*. (b) The second approach assumes that responses are relatively stereotyped and uses a “canonical” response function that entails knowledge about the expected shape of the response. This is preferable, since deconvolution with an uninformed finite impulse response filter poses the risk of overfitting, or taking random fluctuations in the signal as indicative of a meaningful response. Variations in the responses between individuals and conditions can be accounted for by using basis functions derived from Taylor expansions of the canonical response function with respect to its parameters, which is usually time. For example, an SCR slightly shifted in time can be expressed as a linear combination of the canonical response and its derivative with respect to time. In experiment 1, we demonstrate how these derivatives account for variance in the shape of the response, and effectively reduce residual variance. A combination of these basis functions constitutes an “informed basis set”. Note that the approach (a) is the limiting case of an informed basis set that is the least informed allowing for almost any form of the impulse response function.

### 2.2 Constructing a predicted response with a linear convolution filter

In brief, we assume that the SCR time series is the output of an LTI filter, given some specified input functions (see Fig. 1). The input functions will usually be a series of impulses, corresponding to the onsets of specific experimental events. Our prime interest is the amplitude of the response, which is a function of the input under a fixed response function. We can reframe this with a weighting coefficient that is multiplied with the fixed (i.e. standard) input function (e.g. a Dirac delta function per event) and fixed response function. To estimate this weighting coefficient, we construct a predicted time series by convolving the standard input function with the response function, which is defined as a mixture of basis functions. Mathematically, this is the same as a mixture of input functions each convolved with a basis function. The weighting coefficients of this mixture are the parameters of the ensuing general linear model, where the explanatory variables are formed by convolving each input with each basis function and constitute a *design matrix X* (see appendix for details). By inverting this model, we estimate the weighting coefficients and thus, the amplitude of the response.

### 2.3 Statistical inference

We assume as *H*_0_ that an experimental manipulation does not lead to a skin conductance response (i.e. the amplitude of the response is zero), or that two conditions have similar responses (that is, that the difference of their amplitudes equals zero). Inference can be drawn on a within-subject level or between-subjects level. Inference on the within-subject level is affected by autocorrelations in the residuals that render the effective degrees of freedom smaller than the number of data points. One can apply methods to correct the degrees of freedom (as is done in fMRI, see Friston et al., 2008), or use maximum likelihood estimators of the model parameters. This is a non-trivial problem that lies beyond the scope of this paper. Since we are mainly interested in group effects, we only discuss group-level inference that tests for the consistency of responses over individuals. In this case, ordinary least squares parameter estimates on the within-subject level are sufficient for drawing group-level inferences in a summary-statistics approach widely employed in fMRI analyses. For each participant, contrasts or mixtures of parameter estimates of interest are formulated (e.g., the difference between the amplitude of two condition-specific responses). Under the *H*_0_, these will have zero mean which can be tested at the group level using one-sample *t*-tests. An alternative (and mathematically equivalent) approach, used in this paper, is to extract parameter estimates for each individual regressor and use them in a second (between-subject) level ANOVA model, using the partitioned between-subject error variance to test for effects. Arithmetically, both approaches are equivalent to a random effects model given equal variance for different subjects and a balanced experimental design (Friston et al., 2008).

### 2.4 Trial-by-trial variations

Scoring SCRs on a trial-by-trial basis, as in a classical analysis method, allows *post hoc* correlations with trial-by-trial explanatory variables, for example subjective arousal. Post hoc analysis in this form is not possible within the framework described here. However, such relations can be formulated as *a priori* hypotheses and tested within the same model. That is, a regressor can be formed that encodes parametric trial-by-trial variations. To model these parametric effects, the stick function encoding event onsets can be multiplied with any parameter before convolving it with the response function. Mean-centring the resulting regressor ensures it will not explain the average effect of this experimental condition, but only trial-by-trial variations within this condition. The concept of parametric modulators is again derived from fMRI analysis and will be illustrated in experiment 2 and 3.

## 3 Experiment 1

An assumption in our approach is that although response amplitude may vary, the shape of the skin conductance response is constant within one individual and level of experimental factors. Using a canonical basis function posits that responses are sufficiently similar across event types, and between different individuals. Sufficiency, in this framework, means that a major part of the variance can be explained by one response function. Therefore, in the first experiment, we sought to explore within and between subject variability in skin conductance responses. From the responses elicited, we aimed to establish a canonical response function for use in further experiments.

We used loud sounds and aversive pictures to elicit responses. Event onsets were separated by an interval of 30 s or more in order to unambiguously define response tails. Acquired responses were subjected on a trial-by-trial basis to principal component analysis in order to find the response function that would explain maximal variance. For each type of stimulus, 10 responses per participant were obtained, thus providing a dataset of 440 responses across both events and across the whole study sample. We used the whole dataset rather than rejecting “artifacts” and “non-responses” as this makes estimation of the residual variance more conservative. Also, for development of a canonical response, non-systematic noise does not influence the first PCA component and thus leaves the derived response function unaffected.

### 3.1 Methods

#### 3.1.1 Participants and design

The experiment followed a single factorial design with two levels (aversive pictures, aversive sounds). 22 healthy unmedicated participants (11 male, 11 female, mean age ± standard deviation: 22 ± 4.8 years, range 18–34 years) were recruited from the general population and received monetary compensation for their participation. All participants gave written informed consent, and the study was approved by the local ethics committee.

#### 3.1.2 Stimuli and apparatus

Broadband white noise sounds of 1 s length (10 ms onset and offset ramp, ∼95 dB sound pressure level) were delivered via headphones (PX-660 Pro Luxe, Fujikon, Hong-Kong, China). Aversive pictures were drawn from the International Affective Picture System (IAPS; Lang et al., 2005) by using the 10 most arousing negative (valence lower than one standard deviation below the mean for the whole set) pictures and were presented for 1 s. In order to enhance SCR (Boucsein, 1992), stimuli were given additional salience by tasking participants to press the cursor up or down key on a standard computer keyboard to indicate whether they liked the stimulus or not. Stimuli were presented in random order with a maximum of three events of one type in succession. Inter stimulus interval (ISI) between trials was randomly chosen to be 29 s, 34 s, or 39 s, with a mean of 34 s for each participant. The experiment was programmed in Cogent (Version 2000v1.25; www.vislab.ucl.ac.uk/Cogent) on Matlab (Version 6.5; MathWorks; Natick MA; USA), and run on a personal computer with a Pentium 4 processor and a SoundMax soundcard (Analog Devices, Norwood MA, USA).

Skin conductance was recorded on thenar/hypothenar of the non-dominant hand using 8 mm Ag/AgCl cup electrodes (EL258, Biopac Systems Inc., Goleta CA, USA) and 0.5%-NaCl electrode paste (GEL101; Biopac Systems Inc., Goleta CA, USA). In order to avoid motion artefacts (e.g. due to key presses performed with the other hand), we used a motion-restraining armrest. Recordings were conducted in a magnetically shielded room (MSR), using a custom-build constant voltage coupler (2.5 V), based on a differential amplifier and DC-powered by a 12 V battery to minimise electromagnetic noise. The output of the coupler was converted into an optical pulse frequency. This varies sampling rate over time, such that the effective time resolution is determined by the lowest transmission frequency. The lowest sampling rate encountered in any participant was 36.7 Hz. This pulse signal was transmitted using fibre optics, digitally converted outside the MSR with 2 μs time resolution (Micro1401, Cambridge Electronic Design, Cambridge, UK), and recorded (Spike2, Cambridge Electronic Design, Cambridge, UK). Stimulus onset was signalled by TTL pulses of 10 ms length via the stimulus computer's parallel port, and recorded simultaneously with the same time resolution. Temperature and relative humidity of the experimental room were between 18–24 °C, and 30–63%, respectively.

### 3.2 Data analysis

Data analysis was carried out in Matlab (Version 7.4; MathWorks, Natick MA, USA) using custom-made code that is available from the authors. Prior to analysis, skin conductance data were converted back to a waveform signal with 100 Hz time resolution, bandpass filtered with a first order Butterworth filter and cut-off frequencies of 5 Hz, and 0.0159 Hz (corresponding to a time constant of 10 s), respectively, and downsampled to 10 Hz sampling rate. The time-series was then *z*-transformed to account for between-subjects variance in SCR amplitude, which can be due to peripheral and non-specific factors such as skin properties. The 30 s following each event onset were extracted and analysed. Despite filtering, skin conductance level can be different between trials and consequently data from each individual trial was mean-centred. Since SCR might be related to behavioural responses rather than to stimulus onset, we additionally analysed data according to the time point of the button press. This did not explain more variance than an analysis time-locked to stimulus onset, and results from this analysis are omitted for the sake of brevity.

### 3.3 Results

**Fig. 3:**
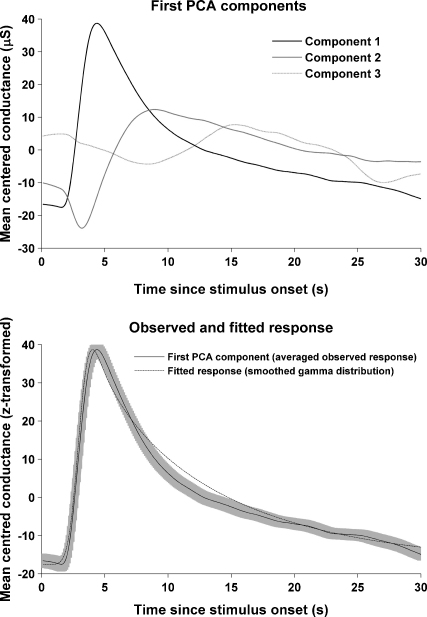
Principal component analysis (PCA) of the responses across all participants, conditions, and trials. Top: the three first components that together explain 78.9% of the overall variability in response shape. Note that the second component resembles a derivative with respect to time of the first component. Bottom: A smoothed gamma distribution (dotted) was fitted to the first component of the PCA (solid line, depicted with standard deviation as grey shadow) to describe the response function's analytical form.

We extracted responses on a trial-by-trial basis and subjected the whole dataset to principal component analysis (PCA) to reduce the number of responses to a set of basis functions that explain the data efficiently. PCA maximises the eigenvalue of the first component, and thus, the first component accounts for the maximum variance that can be explained by one basis function. Results are depicted in Fig. 3. The first component explained 53.2% of the total variance in responses across participants, conditions, and trials. The second and third component explained 19.2% and 6.5%, while further components only explained a minor variance proportion. Note, that the second component resembles an orthogonalised time derivative of the first component.

The residual variance can be further partitioned into between- and within-subjects variance. We accommodated between-subject variance by using one response function per participant, defined as the first PCA component of this participant's responses. This approach explained 67.5% of the total variance. When using one response function for each participant and condition, the first component explained 72.7% of the total variance. This leaves 27.3% within-subject, within-condition variance. Note that this does not only include possible differences in the shape of the response function, but also spontaneous fluctuations of the SCR, and noise due to artefacts associated with the participant or the acquisition equipment.

When identifying a canonical response function across individuals, one can assume that this function is the same across stimulus type or that it is different. To answer this question empirically, we asked if taking into account the averaged response for each condition separately, across all participants, could explain more variance than assuming one general response function. This reduced the unexplained variance by 1.6%, which seemed negligible in the present context.

After showing that a major component of overall variance across individuals and conditions can be explained by a single response function, we sought an analytical form for this function to make it applicable in the analysis of other data sets. A heuristic search over different functional forms revealed a gamma distribution as having a good fit. We smoothed this function in order to render its time derivative useful in accounting for peak shifts, as explained below. The parameters for this function were optimised using a least-square approach (see appendix). The analytical form of this function could account for 52.2% of the overall variance in the original data set which was only slightly less than the first PCA component.

**Fig. 4:**
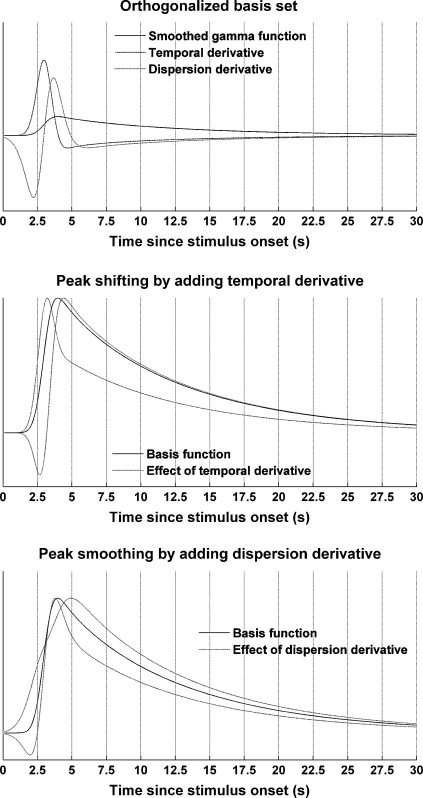
Orthogonalised informed basis set and the effect of adding derivatives to the CRF in order to shift or smooth the response peak.

To reduce residual (error) variance, additional basis functions are often used in fMRI research. Since they are orthogonalised with respect to the canonical response function, they do not change parameter estimates for the canonical response of interest, but increase statistical power by reducing residual variance. In developing such functions, two options are available: one can use empirical PCA components, or in a more analytical fashion, construct derivatives of the canonical response function that account for variations in the timing and shape of the response. Here, we chose the second, more general approach. The response function was differentiated with respect to time and smoothing window size (and thus, peak dispersion). As the derivatives are not orthogonal by nature they were orthogonalised with respect to the response function using a serial Gram-Schmidt procedure. The basis set (canonical, temporal derivative and dispersion derivative) is described in the appendix. Fig. 4 shows the orthogonalised basis set as well as examples of the effect of adding or subtracting derivatives from the basis response function. Using the full basis set, the residual variance was reduced from 48.8% to 35.6%.

A side issue is the impact of high-pass filtering on the response. Filtering is necessary in order to comply with the assumption of a finite response; however, the choice of the filter will necessarily influence the response shape. We applied high-pass filters between 0 and 0.025 Hz in steps of 0.005 Hz and tested their effect on the first PCA component. In bringing the response back to zero within the 30 s interval, filters between 0.005 and 0.015 Hz were most efficient with an optimum at 0.01 Hz. Applying filters gradually reduced the amount of explained variance from 62.1% (no filter) to 56.0%, 54.5%, 53.4%, 53.3%, and 51.5%, respectively, for increasing cut-off frequency. The mutual differences in the response shape (normalised first PCA component) were within two standard deviations of the residuals. This shows that although filtering does influence the response shape, the effect is small compared to the unexplained variance.

### 3.4 Summary

In summary, we analysed skin conductance responses to auditory and visual stimuli in order to assess the intra- and inter-individual variance in these responses. More than 50% of the total variance in mean-centred responses could be explained by the first PCA component (across all trials, conditions, and participants). About 25% of the total variance was caused by within-subjects, within-condition trial-by-trial variations that include spontaneous fluctuations and noise. We conclude it is possible to collapse responses across trials, and to assume a canonical response function across trials, conditions, and participants. Deviations from these assumptions increase error variance and render testing conservative, thus protecting against false positives. We suggest that further investigation can reveal the proportion of variance that stems from noise and spontaneous fluctuations (thus not affecting the invariance assumption) and from variations in response shape.

For an analytical description of the canonical response function, we used a gamma function, convolved with a Gaussian kernel. This function described an almost equal proportion of the variance as the first PCA component. The choice of a smoothed function has a technical justification as both the bi-exponential function favoured by Lim et al. (1997) as well as a raw gamma distribution require parameters that render the derivatives unsuitable for the purpose that they are employed in this paper.

From the canonical response function (CRF), we constructed temporal and dispersion derivatives to reduce between-subject and between-condition error variance. These were orthogonalised to the CRF and thus formed our “informed basis set”. Re-analysis of the data using this basis set drastically reduced residual variance. It should be noted that there was a small advantage in using two different response functions for the two stimulation types, although this seemed negligible in the context of the present experiment. Also, the response functions derived from this experiment should be applied with a little caution to experimental paradigms that are different from the ones used here. In addition, we show that the filter settings have a small impact on the shape of the response function. Therefore, it might be important to use the same filter settings for analysis that were used for the development of the response function. Future work will explore optimal filter settings in this analysis framework. For the present paper, we continued to use a high-pass filter of 10 s time constant as this is the most well-established setting in the literature.

## 4 Experiment 2

The aim of experiment 2 was to test the utility of our method in the analysis of SCRs in a short ISI paradigm using the CRF derived from experiment 1. Additionally, we sought to investigate the use of parametric modulators to account for habituation of responses. To achieve these goals, we repeatedly elicited SCRs to 95 dB white noise bursts of 1 s length and varied mean ISI in a block-wise fashion (3 s, 9 s, 19 s).

### 4.1 Methods

#### 4.1.1 Participants and design

The experiment followed a single factorial design with three levels of the mean ISI (3 s, 9 s, and 19 s). 24 healthy unmedicated participants (12 male, 12 female, mean age ± standard deviation: 27 ± 4.6 years) were recruited from the general population and received monetary compensation for participation. The sample was completely independent from experiment 1, and identical with experiment 3. All participants gave written informed consent, and the study was approved by the local ethics committee.

#### 4.1.2 Stimuli and apparatus

In each of the three conditions, as defined by mean ISI, 15 broadband white noise sounds of 1 s length (10 ms onset and offset ramp, ∼95 dB sound pressure level) were delivered via headphones (PX-660 Pro Luxe, Fujikon, Hong-Kong, China). Participants were instructed to press a button on a standard computer keyboard as quickly as possible when they heard a sound. Mean ISI was varied block wise. Blocks were separated by additional intervals of 15 s, and the six possible block orders were balanced across participants. The ISI was randomly jittered within ±30% of the mean ISI. The experiment was programmed in Cogent (Version 2000v1.25; www.vislab.ucl.ac.uk/Cogent) on Matlab (Version 6.5.; MathWorks; Natick MA; USA), and run on a personal computer with a Pentium 4 processor and a SoundMax soundcard (Analog Devices, Norwood MA, USA). Skin conductance recordings were obtained as described in experiment 1. The lowest sampling rate encountered in any participant was 31.6 Hz. Temperature and relative humidity of the experimental room were between 20°–25°, and 39%–62%, respectively.

Experiment 2 and 3 were conducted on the same sample. Since no direct comparison between the experiments was intended and the paradigm in experiment 2 was assumed to be less susceptible to habituation, experiment 2 always followed experiment 3 after a short break.

#### 4.1.3 Data analysis

Data analysis was carried out in Matlab (Version 7.4; MathWorks, Natick MA, USA) using custom-made code that is available from the authors. Prior to analysis, skin conductance data were pre-processed as described in experiment 1.

### 4.2 Results

**Fig. 5:**
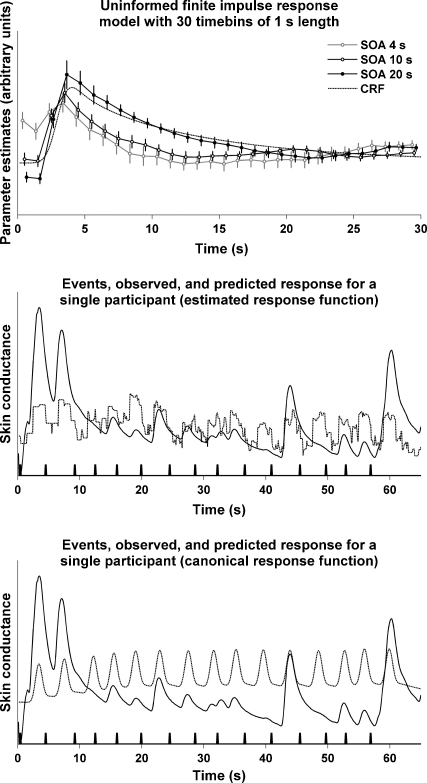
Experiment 2: Example of estimating the response shape, using an uninformed finite impulse response model that consists of a number of boxcar functions for each time bin during the response. Here, we used 30 post-stimulus time bins of 1 s length. Top: Fitted responses across the study sample for the three different ISIs, mean ± standard error across participants. For comparison, the broken line depicts the canonical response function (CRF), derived from experiment 1. Middle: Continuous data for one participant at an ISI of 3 s. Event onsets are marked on the *x*-axis. The observed and predicted responses are shown as solid and broken lines, respectively. Bottom: Similar to the middle panel, this panel shows observed and predicted responses for the same participant, using the canonical response function and its two derivatives. Note that for comparison, this model did not include adaptation parameters. Adaptation parameters further improved the model fit, as pointed out in the text.

In a first step, we analysed the SCR time-series with different regressors for each ISI, using an uninformed finite impulse response filter of 30 s length and time bins of 1 s. Thus, the averaged SCR could be estimated for each condition with a time resolution of 1 s. Fig. 5 (top panel) shows the results and demonstrates that the filter effectively deconvolves the time-series even at short ISIs. That is, although the peak of each response extends into the following events, the shape of the averaged response can be isolated. However, the estimated response shape differed between the ISIs (*F*_72, 576_ = 2.6; *p* < .0001), an effect that could be due to estimation imprecision or to real differences due to the stimulus repetition rate. Also, it also can be seen in Fig. 5 (top panel) that the model using an uninformed finite impulse response filter is not particularly effective in estimating the tail of the response. Therefore, a method that imposes more constraints on the shape of the expected response might be preferable.

Here, we used the informed basis set derived from experiment 1 to create a second model and to estimate the amplitude of the canonical response function (and its derivatives) for responses at each ISI. As SCRs can decrease over time and consequently to demonstrate how this can be tested within the present framework, we included an additional regressor for each ISI that modelled a linearly decreasing effect of time. This entailed a parametric modulation of the input function and convolution with the canonical response function. This regressor was orthogonalised with respect to the main regressor for each block, and in addition we included orthogonalised time and dispersion derivatives for each regressor. Parameter estimates scaling the canonical response function were extracted from each participant and analysed on the second level with a one way ANOVA model with the factor ISI and *post hoc* one-sample *t*-tests. This revealed a significant response at each ISI, and a significant effect of a time-dependent decrease of SCR across all ISIs (all *p* < .001). The overall response was larger at longer ISIs (*F*_2, 46_ = 31.0; *p *< .001), while there was no significant effect of ISI on adaptation (*F*_2, 46_ = 1.4; *p* > .25). To compare the implicit condition-specific SCR functions under the uninformed finite impulse response and the canonical convolution models, Fig. 5 (bottom panel) shows the estimated responses based on the informed model. These are simply the mixture of informed basis functions weighted by the least squares parameter estimates. It can be seen that the uninformed finite impulse response (middle panel) and the informed basis set (bottom panel) give similar results but the informed estimates are regularised and smoother.

### 4.3 Summary

The aim of experiment 2 was to demonstrate the use of the linear deconvolution method for the detection of SCRs at short ISIs. In a first analysis, an uninformed finite impulse response model was used. Thus, we show that deconvolution of responses is possible even at short ISIs. However, the shape of the estimated response differed with ISI. This might reflect non-linearities in the summation of SCRs or to over-fitting of the filter. To preclude over-fitting, we used a convolution model based on the canonical response function and its derivatives from experiment 1 and were able to detect responses at all ISIs, and to demonstrate adaptation (i.e., the physiological homologue of behavioural habituation) within the blocks.

While adaptation was independent of ISI, responses were higher at longer ISIs which suggests that SCRs to successive stimuli that occur rapidly are attenuated in relation to equivalent responses to stimuli that are presented sparsely. This might imply that non-linear saturation is a feature of SCRs when presented in fast succession. We show however that under that the assumption of linearity, SCRs can be de-convolved and effectively analysed. Thus, we have shown with this example that these putative nonlinearities can be modelled perfectly well with a linear convolution model provided ISI-dependent effects are included explicitly.

In conclusion, we have demonstrated that it is possible to deconvolve skin conductance time-series at short ISIs, and that the use of a canonical response function facilitates detection of responses and time-dependent changes in this response, such as adaptation. Furthermore, even if there are nonlinear interactions between stimuli occurring in quick succession, these can be modelled using a linear convolution model that incorporates ISI as an experimental factor. This experiment however tested SCRs in a rather simple paradigm and therefore does not allow a statement as to whether this method would also be useful in more complex designs. In experiment 3 we address such a design.

## 5 Experiment 3

Here, we used our method in a complex paradigm with emotional stimuli implemented by presenting aversive and neutral pictures at different ISIs, enabling us to investigate whether effects depended on ISI. In addition, we applied a classical peak-scoring analysis to directly compare its power to detect condition-specific differences with our deconvolution approach.

### 5.1 Participants and design

The experiment used a 2 × 3 factorial design with the factors picture type (aversive, neutral), and mean ISI (3 s, 9 s, and 19 s). It was conducted on the same sample (*N* = 24) and preceded experiment 2.

### 5.2 Stimuli and apparatus

Stimuli were drawn from the International Affective Picture System (IAPS; Lang et al., 2005) using the 90 most arousing and negative (valence lower than one standard deviation below the mean for the whole set) pictures as aversive stimuli, and the 90 least arousing neutral (valence within mean ± standard deviation for the whole set) pictures as neutral stimuli. Each of the two picture types was randomly assigned to three aversive and three neutral sets with similar arousal ratings, respectively.

The assignment of the three aversive and the three neutral sets to ISI levels was balanced across participants. Neutral and aversive stimuli were randomly interleaved for each participant. Pictures were presented for 1 s. Stimulus onset was randomly varied around the mean ISI with a jitter of 0.08 s, 0.68 s, and 1.68 s, respectively. Participants were tasked to press the cursor up or down key of a standard computer keyboard to indicate whether they liked the stimulus or not. Skin conductance recordings were obtained as described in experiment 1. The lowest sampling rate encountered in any participant was 30.5 Hz.

### 5.3 Data analysis

Data analysis was carried out in Matlab (Version 7.4.0; MathWorks, Natick MA, USA) using custom-made code that is available from the authors. Prior to analysis, skin conductance data were pre-processed as described in experiment 1. For each of the six event types, a stick function encoding event onsets was convolved with the canonical skin conductance response function derived from experiment 1. Orthogonalised temporal and dispersion derivatives were included as separate regressors to account for between-subject and between-conditions variance in response shape. Subjective dichotomous likeability ratings were included as additional parametric regressors for each event type, orthogonalised to the main regressor and its derivatives, to account for variance within the event type. To account for adaptation of responses, additional regressors were included that modelled a linear decrease of responses over all trials. Parameter estimates for the canonical response function were extracted for each participant, and together with behavioural measures of response accuracy, likeability rating and reaction times, tested at the group level in SPSS (Version 12; Chicago IL, USA). Greenhouse-Geisser correction for degrees of freedom was used in all repeated measures ANOVAs.

For comparative purposes, skin conductance data were also analysed using a classical peak-scoring approach that extracted the maximum amplitude between 0 and 5 s after event onset and corrected for a baseline between 0.5 and 0 s before event onset, either thresholding peaks at 0.1 μS or not thresholding. A further analysis was conducted using an analytical form of the method proposed by Barry et al. (1993). The first derivative of the skin conductance time-series was smoothed over a time window of ±1 s around each event onset and was used to construct a tangent to the skin conductance time-series through the event onset. This tangent was then used to correct the time series, and from the corrected time series, the maximum was extracted as described above, either thresholded or unthresholded.

Using in house software we also reproduced the methods described by Lim et al. (1997) and Alexander et al. (2005). To emulate the approach of Lim et al., we re-sampled the signal to 1/256 s time resolution, cut out 10 s epochs following each event and fitted the six-parameter function (using gradient search and a least square error criterion). This provided estimates of peak latency and amplitude. For the Alexander method, we re-sampled the signal to 0.02 ms time resolution, integrated and smoothed the estimated driver function and its derivative. We then estimated the threshold *P* for peak detection from the data. No peaks could be identified in any dataset. We did not pursue this approach further.

### 5.4 Results

#### 5.4.1 Reaction times, accuracy and likability

Behavioural measures are listed in Table 1. Aversive pictures were less likely to be missed and to be rated as likeable, and reaction times were quicker. Reaction times were longer when the ISI was longer.

#### 5.4.2 Skin conductance responses

Parameter estimates for the different regressors are summarized in Table 2. Parameter estimates for the CRF deviated from zero across conditions and participants, as shown by a significant intercept, indicating that SCRs could be detected. Parameter estimates were dependent on ISI, with higher parameter estimates at longer ISIs. As expected, aversive pictures yielded higher parameter estimates than neutral pictures, while this differential effect was not dependent on ISI. In addition, we show a significant trial-by-trial correlation of likeability ratings with the skin conductance responses within all conditions. This effect was not dependent on picture type or ISI. Note that this analysis accounted only for variance in likeability ratings within conditions, not between conditions.

Using the alternative approaches, we could demonstrate that SCRs were elicited in response to picture viewing, but there was no effect of picture type or ISI in any of the analyses.

### 5.5 Summary

In an emotional picture viewing paradigm, we show evoked SCRs and an effect of ISI on SCR amplitude. We observed higher responses to aversive than to neutral pictures, a differential effect not dependent on ISI, indicating the power of our model to detect condition differences even at very short ISIs. Subjective likeability ratings were associated with skin conductance responses within all conditions and ISIs. This is in line with previous studies using longer ISIs (Amrhein et al., 2004).

To demonstrate that our algorithm is more powerful than previous methods, we analysed results using a peak-to-baseline scoring method, a baseline correction method similar to the one proposed by Barry et al. (1993), and the 6-parameter model of Lim et al. (1997). In these analyses, SCRs could be detected, but no differential effect of picture type was observed. We failed to detect any responses with the approach of Alexander et al. (2005) due to its problematic handling of measurement noise.

## 6 Discussion

In this paper we propose a novel approach to the analysis of evoked skin conductance responses that resolves the problem of overlapping responses encountered in paradigms involving short stimulus onset asynchronies (ISI). This approach is based on modern methods for analysing blood oxygen level dependent (BOLD) responses in functional magnetic resonance imaging, where similar issues arise. Beyond practical purposes, its main advantage is the analytical description of the underlying assumptions that posit a linear time invariant system as generator of SCRs.

In our experiment involving SCRs to loud white noise bursts and aversive pictures, we show that just one response function can explain the major proportion of the within- and between-subject variance. Hence, we developed a skin conductance canonical response function. Time and dispersion derivatives of the CRF were then used to account for inter-individual differences and explained a significant amount of additional variance. In a second experiment we use this CRF and show that the method is capable of de-convolving SCRs to loud white noise bursts at ISIs as short as 3 s, and of testing *a priori* hypotheses about parametric trial-to-trial effects on SCRs, in this case adaption. In a third experiment we demonstrate that differences in SCRs in response to aversive and neutral pictures, as well as modulation of responses by subjective ratings, can be detected at a range of ISIs. Comparative evaluations also suggested that our method is superior to other peak-scoring algorithms, which for the present dataset could not detect an effect of picture valence.

We conclude that our model is viable for analysing SCR in short ISI paradigms. However, several limitations need to be taken into account. At the ISIs used in the present study (that is, 3–19 s), the response seems to be dependent on ISI, thus pointing to non-linearities. This suggests that stimuli presented at different ISIs should be modelled as different levels of an experimental (ISI) factor and that other factors may interact with ISI. Future work will explore this, as well as the impact of stimulus type on shape and time-invariance of SCRs. In addition, it would be preferable to relate SCR shape not only to experimental input, but also to sudomotor nerve activity as e.g. measured by microneurography (Macefield and Wallin, 1996), and sweat gland opening as revealed by videomicroscopy (Nishiyama et al., 2001). Such experiments might lead to a better understanding of the methods presented here. A second limitation is the utility of the approach for detecting whether responses are elicited to a repeated stimulus where long ISI paradigms offer greater power for statistical reasons, as the predicted time-series contains more variance than at short ISIs (Friston et al., 2008). This is well recognised in fMRI research. However, for detecting condition differences, it has been shown that in fMRI, short ISIs offer greater power, as more events of each type can be realised. Experiment 3 aimed at testing such differences in a paradigm with two interleaved event types. We could show that the difference between event types was not dependent on ISI, thus making short ISI designs potentially more efficient.

The analytical form of our model has the advantage that all assumptions are made explicit. This may be perceived as implying that more assumptions are made in our model than in classical SCR analysis. Such an impression is misleading as classical methods also make assumptions, albeit without describing or testing them. As an example, the assumption that the response peak or the area under the curve reflects precise estimates of the input signal only pertains when the response shape is constant. When response shape varies, this assumption needs to be explicitly stated and justified. In contrast, the assumptions of our model are stated in fully analytical terms and are therefore easy to test using model comparison. Also, our model can be expanded to deal with less strict assumptions. For example, if the linearity assumption is violated, one could use a non-linear convolution model based on Volterra kernels (see Friston et al., 1998 for an application in fMRI).

To conclude, we present a novel approach to the analysis of event-related SCRs and show its efficiency in detecting responses and condition-specific differences of these responses at long and short ISIs, and therefore offers an alternative to classical approaches. Our model makes explicit assumptions that are sufficiently met for practical implementation and can be further validated using model comparison. As our method uses all available information, instead of just one number per event as in classical methods, it may be more powerful and offer greater experimental flexibility than previous approaches to SCR analysis.

## Figures and Tables

**Table 1:** Experiment 3: Behavioural results (mean ± standard error of the mean) for the six experimental conditions. Accuracy refers to the number of responses made per condition (in %). We report uncorrected degrees of freedom, *F*-values, *ɛ*-values according to Greenhouse-Geisser, and corrected *p*-values.

	ISI	3 s	9 s	19 s	Picture type	ISI	Interaction
		Mean ± SEM	Mean ± SEM	Mean ± SEM	*F*(1, 23) *ɛ*	*F*(2, 46) *ɛ*	*F*(2, 46) *ɛ*
Accuracy (%)	Neutral	99.4 ± 0.6	99.4 ± 0.3	97.9 ± 0.66	8.4^**^	3.2	1.3
	Aversive	99.8 ± 0.1	99.6 ± 0.4	99.4 ± 0.33	1	0.591	0.893

Rating as likeable (%)	Neutral	73.3 ± 2.8	76.0 ± 3.9	74.7 ± 3.6	531.9^***^	<1	<1
	Aversive	6.7 ± 2.2	6.1 ± 2.2	5.9 ± 1.3	1	0.946	0.830

Reaction time (ms)	Neutral	1008 ± 34	1063 ± 48	1118 ± 50	26.7^***^	7.6^**^	<1
	Aversive	858 ± 38	920 ± 52	970 ± 51	1	0.963	0.990

^**^p < .01; ^***^p < .001.

**Table 2:** Experiment 3: Parameter estimates (arbitrary units) for skin conductance responses (mean ± standard error of the mean) for the six experimental conditions. In the present model, a significant intercept refers to the fact that parameter estimates deviate from zero, and shows that an effect was detected across all experimental conditions. (Estimated) SCR peak amplitude in the lower part of the table is reported in μS (mean ± standard error of the mean). The table states uncorrected degrees of freedom, *F*-values, *ɛ*-values according to Greenhouse-Geisser, and corrected *p*-values.

	ISI	3 s	9 s	19 s	Intercept	Picture type	ISI	Interaction
		Mean ± SEM	Mean ± SEM	Mean ± SEM	*F*(1, 23)	*F*(1, 23) *ɛ*	*F*(2, 46) *ɛ*	*F*(2, 46) *ɛ*
SCR (parameter estimate)	Neutral	−1.1 ± 6.2	19.0 ± 12.0	40.5 ± 15.0	6.3*	5.1*	6.1*	<1
	Aversive	11.8 ± 5.7	22.2 ± 13.4	50.0 ± 16.5		1	0.722	0.818

Effect of stimulus rating on SCR (parameter estimate)	Neutral	16.6 ± 10.7	0.8 ± 12.8	0.3 ± 8.0	5.2*	1.3	<1	<1
	Aversive	20.1 ± 15.3	20.4 ± 12.8	19.8 ± 22.9		1	0.968	0.899

SCR (peak-scoring)	Neutral	0.015 ± 0.003	0.014 ± 0.005	0.014 ± 0.005	16.6**	<1	<1	1.6
	Aversive	0.018 ± 0.006	0.009 ± 0.003	0.012 ± 0.004		1	0.990	0.853

SCR (according to Barry et al., 1993)	Neutral	0.033 ± 0.005	0.027 ± 0.004	0.005 ± 0.029	51.9**	<1	2.0	2.2
	Aversive	0.030 ± 0.004	0.027 ± 0.004	0.029 ± 0.005		1	0.862	0.977

SCR (according to Lim et al., 1997)	Neutral	0.7 ± 0.4	0.3 ± 0.1	0.3 ± 0.1	6.2*	2.4	1.4	1.6
	Aversive	1.9 ± 0.9	0.7 ± 0.3	0.3 ± 0.1		1	0.565	0.690

* *p* < .05.

** *p* < .01.

## Appendix A

The assumption made in this paper is that the skin conductance response *s*(*t*) is the output of a finite Linear Time-Invariant filter:

• The linearity property satisfies the superposition principle that the response at a given time caused by two or more stimuli is the sum of the responses which would have been elicited by each stimulus individually.
• The time-invariant property means that the output does not explicitly depend on time, which corresponds here to say that the impulse response function is not a function of time except expressed by input.
• The finite property states that the response settles to zero after a finite interval.

A LTI filter is entirely characterised by its impulse response function *h*(*t*) in such a way that the SCR is simply the convolution of the input signal *u*(*t*) with the impulse response function that models the time delay of the skin conductance response and its specific shape

$$s(t)=h(t)⊗u(t)=\int_{-\infty }^{+\infty }h(t-\tau )u(\tau )\;\text{d}\tau$$

Note that the convolution is a linear operator.^1^

Deconvolution is the process of filtering the observed output signal *s*(*t*) to recover the input signal *u*(*t*) by removing the effect of the convolution filter *h*(*t*).This is an ill-posed problem even in the absence of observation noise, thus requiring some form of regularisation.

In the following we will assume that the impulse response function *h*(*t*) is known (estimated from a principal component analysis on an independent dataset) and we will call it the *canonical response function* ${\tilde{h}}_{can}(t)$, or CRF. We assume that it has a parametric form using a Gaussian smoothed probability density function of a Gamma distribution:

$${\tilde{h}}_{can}(t)=N(t)⊗G(t)$$

where *N*(*t*) is a centered Gaussian function with a standard deviation *σ* of 0.5657 s.

$$N(t)=\frac{1}{\sqrt{2\pi }\sigma }\;{\text{e}}^{-t^{2}/2\sigma^{2}}$$

and *G*(*t*) is a probability density function of a Gamma distribution:

$$G(t)=\frac{l^{h}{\left(t-t_{0}\right)}^{h-1}\;{\text{e}}^{-l(t-t_{0})}}{\Gamma (h)}$$

for *t > t*_0_ and 0 otherwisewhere the parameters *h* and *l* describe the shape and the inverse scale (rate) of the distribution, respectively, *t*_0_ is the delay of the response and *Γ* is the Gamma function defined as:

$$\Gamma (x)=\int_{0}^{\infty }t^{x-1}\;{\text{e}}^{-t}\;\text{d}t$$

In practice, the Gaussian function was truncated within a centered window of 4 s and the CRF was specified for the time interval of 0–120 s and was set to 0 everywhere else.

The parameters of this analytical form of the CRF are estimated using the dataset presented in experiment 1 of this paper using a least-square approach. The estimated parameters are:

$$\left\{\begin{array}{l} \hat{h}=0.9445\\ \hat{l}=0.1037\\ {\hat{t}}_{0}=2.9971 \end{array}\right)$$

To take into account that this *canonical* response might not fit equally well the actual impulse response function for different conditions or individuals, it is possible to define a constrained basis set spanning the space of possible responses: this is called an *informed basis set* ${\left\{{\tilde{h}}_{\inf}^{i}(t)\right\}}_{i=1\ldots N}$. One way to construct this basis set is to perform a multivariate first-order Taylor expansion of the canonical response along parameters that are expected to vary between experimental conditions or subjects. Here we will consider time and dispersion parameters. Thus, for the construction of the temporal derivative $\partial {\tilde{h}}_{can}/\partial t$, the canonical function is differentiated with respect to time. The dispersion derivative $\partial {\tilde{h}}_{can}/\partial \sigma$ is obtained by computing the difference between the original CRF and the same one convolved with a wider Gaussian window (8 s duration and a standard deviation of 1.1314 s).

The *informed basis set* capturing the CRF is therefore:

$${\left\{{\tilde{h}}_{\inf}^{i}(t)\right\}}_{i=1\ldots 3}=\left\{{\tilde{h}}_{can},\frac{\partial {\tilde{h}}_{can}}{\partial t},\frac{\partial {\tilde{h}}_{can}}{\partial \sigma }\right\}$$

This basis set was orthogonalised using a serial Gram-Schmidt process as implemented in the SPM software (spm_orth.m). This does not change the space spanned by the basis set but will make sure that the effect explained by the canonical response is not partialed out by the other regressors of the basis set.

The impulse response function for a particular dataset can now be appropriately modelled by a linear combination of the functions comprised in the informed basis set.

The skin conductance response *s*(*t*) with *M* experimental conditions can then be expressed as

$$s(t)=\sum_{m=1}^{M}\sum_{n=1}^{N}\beta_{mn}{\tilde{h}}_{\inf}^{n}⊗{\tilde{u}}_{m}(t)$$

where ${\tilde{u}}_{m}(t)$ is a time-dependent input parameter of interest (e.g. an event onset function) with unit amplitude for condition *m*, defined as a sum of Dirac delta functions centered on the onsets ${\left\{\tau_{k}^{M}\right\}}_{k=1\ldots K}$ of the events of condition *m*

$${\tilde{u}}_{m}(t)=\sum_{k=1}^{K_{m}}\delta (t-\tau_{k}^{m})$$

and *β* is a vector containing the weights of the linear combination.

Note that this framework allows to model parametric modulations such as time (adaptation effect) or any other factors and any non-linear effect of those using polynomial expansion. For example, if ${\left\{p_{k}\right\}}_{k=1\ldots K_{m}}$ is such a parametric modulator, defined for all events, then another input parameter can be defined by

$$\sum_{k=1}^{K_{m}}p_{k}^{2}\delta (t-\tau_{k}^{m})$$

to model quadratic interactions between the variate *p* and the trials.

All of this can be rephrased in terms of a general linear model (GLM): let *Y* contain *T* observed datapoints of a skin conductance time-series and *X* a *T* × *L* matrix containing *L* regressors, called the *design matrix*, constructed as explained above (convolution of all the input functions with the informed basis set), then the GLM can be expressed as

Y=Xβ+ε

where *β* is defined as before (a *L* × 1 vector of weights or effect sizes) and *ɛ* is the error term that is assumed to be i.i.d. (independent and identically distributed) Gaussian noise.

Under these assumptions, the maximum likelihood estimators are obtained through the Gauss-Markov theorem and are equal to the ordinary least square estimates (OLS):

$$\hat{\beta }={\left(X^{T}X\right)}^{-1}X^{T}Y$$

Note that this is a linear operation: the estimates $\hat{\beta }$ are obtained through a linear combination of the data *Y*.

If *X* is not of full rank, the model can still be inverted using the Moore-Penrose pseudoinverse.

Inverting a GLM can then be seen as a way to perform linear deconvolution. If the basis set is chosen to be a series of boxcar functions for each time bin, then no assumption is made on the shape of the impulse response function (apart from its length). If some constraints are added by using an informed basis set (restricting the domain of variations spanned the response to some plausible ones) then it still corresponds to some deconvolution that will likely provide some more robust estimates of the response. The extreme case is to only use the canonical response and in such a case, the response is assumed to be known and only the amplitude of the response remains to be estimated.

At last, once inverted, it is possible to perform some statistical inference on the estimated parameters to, for example, compare the effect sizes elicited by two conditions, using *T* or *F* tests. This can be done on the group level using a hierarchical summary statistics approach which is equivalent to a random effects model given equal variance for different subjects and a balanced experimental design (Friston et al., 2008).
